# Anti-Inflammatory Effect of the Blueberry Anthocyanins Malvidin-3-Glucoside and Malvidin-3-Galactoside in Endothelial Cells

**DOI:** 10.3390/molecules190812827

**Published:** 2014-08-21

**Authors:** Wu-Yang Huang, Ya-Mei Liu, Jian Wang, Xing-Na Wang, Chun-Yang Li

**Affiliations:** 1 Department of Functional Food and Bio-active compounds, Institute of Farm Product Processing, Jiangsu Academy of Agricultural Sciences, Nanjing 210014, China; E-Mails: wuyanghuang@jaas.ac.cn (W.-Y.H.); wangjian454348952@126.com (J.W.); wangxingna@jaas.ac.cn (X.-N.W.); 2 National Technical Research Centre of Veterinary Biological Products, Jiangsu Academy of Agricultural Science, Nanjing 210014, China; E-Mail: yameiliu@jaas.ac.cn; 3 College of Food Science and Technology, Nanjing Agricultural University, Nanjing 210095, China

**Keywords:** anthocyanins, anti-inflammatory effect, blueberry, malvidin-3-galactoside, malvidin-3-glucoside

## Abstract

Blueberry fruits have a wide range of health benefits because of their abundant anthocyanins, which are natural antioxidants. The purpose of this study was to investigate the inhibitory effect of blueberry’s two main anthocyanins (malvidin-3-glucoside and malvidin-3-galactoside) on inflammatory response in endothelial cells. These two malvidin glycosides could inhibit tumor necrosis factor-alpha (TNF-α) induced increases of monocyte chemotactic protein-1 (MCP-1), intercellular adhesion molecule-1 (ICAM-1), and vascular cell adhesion molecule-1 (VCAM-1) production both in the protein and mRNA levels in a concentration-dependent manner. Mv-3-glc at the concentration of 1 μM could inhibit 35.9% increased MCP-1, 54.4% ICAM-1, and 44.7% VCAM-1 protein in supernatant, as well as 9.88% MCP-1 and 48.6% ICAM-1 mRNA expression (*p* < 0.05). In addition, they could decrease IκBα degradation (Mv-3-glc, Mv-3-gal, and their mixture at the concentration of 50 μM had the inhibition rate of 84.8%, 75.3%, and 43.2%, respectively, *p* < 0.01) and block the nuclear translocation of p65, which suggested their anti-inflammation mechanism was mediated by the nuclear factor-kappa B (NF-κB) pathway. In general malvidin-3-glucoside had better anti-inflammatory effect than malvidin-3-galactoside. These results indicated that blueberry is good resource of anti-inflammatory anthocyanins, which can be promising molecules for the development of nutraceuticals to prevent chronic inflammation in many diseases.

## 1. Introduction

**Figure 1:**
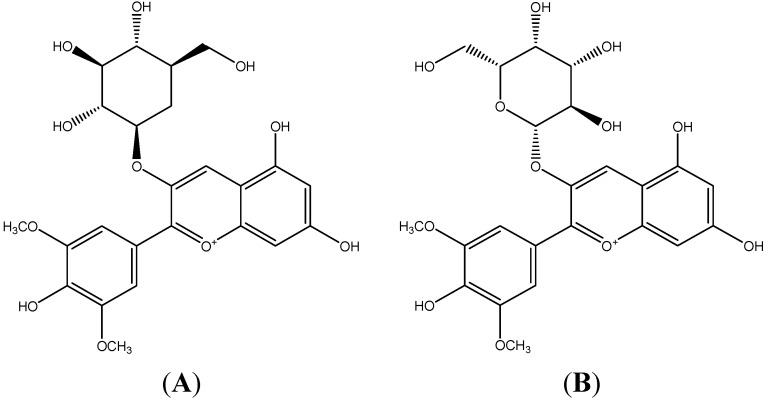
The structures of malvidin-3-glucoside (**A**) and malvidin-3-galactoside (**B**).

Due to their antioxidant capacity and nutritional quality fruits are an economical potential resource of functional foods and nutraceuticals, so they play an important role in human nutrition and health [1,2]. Fruits possess diverse phenolic compounds (e.g., phenolic acids and polyphenols which include flavonoids (anthocyanins, flavanols, and catechins) and tannins), carotenoids, and vitamin C and E, which are well-known dietary antioxidants beneficial to the endogenous antioxidant defense strategies [3]. These bioactive phytochemicals contribute the major health-promoting function of fruits [4,5]. Rabbiteye blueberry (*Vaccinium ashei*) has a wide range of bioactivities, such as super antioxidant ability, anti-diabetic capacity, anti-proliferative quality, anti-inflammatory effects, and protective effects protect against cancer and stroke [6,7]. Besides, blueberries may alleviate conditions occurring in Alzheimer’s disease and aging [8]. Blueberries also help to maintain healthy blood flow via LDL oxidation, normal platelet aggregation, and endothelial function improvement [9,10]. Anthocyanins in blueberries are mainly responsible for those health benefits [11]. Accumulation of reactive oxygen species generated by inflammatory cells is thought to be one of the major factors contributing to chronic inflammation in many diseases, such as atherosclerosis [12], so antioxidants could be considered to have potential capacity to prevent and treat chronic inflammation. Compared with the synthetic antioxidants, natural antioxidants are higher efficient and economical. Besides, synthetic antioxidants may exhibit toxicity and side-effects. Thus, it’s urgent to search for more natural antioxidant resources. Anthocyanins are considered as most potent natural hydrophilic antioxidants and their properties extend well beyond suppressing free radicals [13]. According to an investigation at Tufts University in the USA, the content of anthocyanins in blueberry was reported as the highest among 40 vegetables and fruits [14]. We previously demonstrated the presence of nine anthocyanins in blueberry and found that malvidin-3-glucoside (Mv-3-glc) and malvidin-3-galactoside (Mv-3-gal) were the most abundant [15]. Their structures are shown in Figure 1. In addition, malvidin has been found to possess inhibition to TNF-α-induced inflammatory responses [16], and malvidin-3-glucoside in wild blueberries significantly reduces the expression of pro-inflammatory genes *in vitro* [17]. Potentiating interactions can be additive, concommitant, inhibitory, supraadditive, or synergistic. In light of the observed synergism of antioxidants, such as vitamin E and vitamin C, catechin and malvidin 3-glucoside, and tea polyphenols and vitamin E [18,19], it has been suggested that combinations of antioxidants with different radical scavenging efficiency may show a different antioxidant activity from the expected one based on the sum of their individual effects [20]. In the present study, we further investigated the anti-inflammatory properties of Mv-3-glc and Mv-3-gal, as well as their synergistic effect in human vascular umbilical endothelial cells.

## 2. Results and Discussion

### 2.1. Effect of Mv-3-glc and Mv-3-gal on TNF-α-Induced MCP-1, ICAM-1, and VCAM-1 Production in Supernatant

As shown in Table 1, the surface protein expression of MCP-1, ICAM-1, and VCAM-1 was low in unstimulated cells. Exposure of cells to TNF-α (10 μg/L) for 6 h significantly induced up-regulation of surface expression of MCP-1, ICAM-1, and VCAM-1, although expression levels were not same for Mv-3-glc, MCP-1 protein expression increased from 0.023 μg/L to 0.519 μg/L, ICAM-1 increased from 0.111 μg/L to 0.605 μg/L, and VCAM-1 increased from 0.021 μg/L to 0.314 μg/L. For Mv-3-gal, MCP-1 increased from 0.023 μg/L to 0.264 μg/L, ICAM-1 increased from 0.102 μg/L to 0.337 μg/L, and VCAM-1 increased from 0.013 μg/L to 0.124 μg/L. For Mv-3-glc and Mv-3-gal mixture, MCP-1 increased from 0.015 μg/L to 0.507 μg/L, ICAM-1 increased from 0.161 μg/L to 0.625 μg/L, and VCAM-1 increased from 0.014 μg/L to 0.445 μg/L. Pretreatment with Mv-3-glc, Mv-3-gal and their mixture partially inhibited this up-regulation in a concentration-dependent manner. In most time, the inhibitory effect of Mv-3-glc was better than Mv-3-gal. Mv-3-glc at the concentration of 1 μM could inhibit 35.9% increased MCP-1 production (0.341 μg/L), 54.4% increased ICAM-1 (0.336 μg/L) and 44.7% increased VCAM-1 (0.183 μg/L) (all *p* < 0.01), while 1 μM Mv-3-gal could only inhibit 18.2% MCP-1 (0.216 μg/L) (*p* < 0.05), 54.9% ICAM-1 (0.208 μg/L) and 19.9% VCAM-1 (0.106 μg/L) (both *p* < 0.01). Mv-3-glc at the concentration of 10 μM could inhibit 66.3% increased MCP-1 production (0.175 μg/L) and 63.8% increased VCAM-1 (0.127 μg/L) (both *p* < 0.001), and 69.6% increased ICAM-1 (0.261 μg/L) (*p* < 0.01), while 10 μM Mv-3-gal could inhibit 64.3% MCP-1 (0.109 μg/L), 52.3% VCAM-1 (0.066 μg/L), and 64.7% ICAM-1 (0.185 μg/L) (all *p* < 0.01), respectively. In addition, 50 μM and 100 μM Mv-3-glc both inhibited more than 90% increased three protein expressions (*p* < 0.001). The Mv-3-glc and Mv-3-gal mixture at the concentration of 10 μM inhibited 86.8% increased MCP-1 (0.080 μg/L), 98% increased ICAM-1 (0.170 μg/L), and 70.1% increased VCAM-1 (0.143 μg/L) (all *p* < 0.001), which was greatly stronger than Mv-3-glc and Mv-3-gal respectively. This indicated that Mv-3-glc might have additive effect with Mv-3-gal. For ICAM-1, all the three treatments at different concentrations got the inhibition rate more than 50%. Even, 1 μM Mv-3-glc and Mv-3-gal mixture could inhibit more than 90% increased ICAM-1. ICAM-1 expression was less than the control when cells were treated with high concentration of these two malvidin glycosides, which indicated that they might directly decrease ICAM-1 production without stimulation of TNF-α.

**Table 1:** Effects of Mv-3-glc and Mv-3-gal on TNF-α-induced MCP-1, ICAM-1, and VCAM-1 in supernatant.

Treatment	Protein in Supernatant (μg/L)		
	MCP-1	ICAM-1	VCAM-1
Control	0.023 ± 0.001 ***	0.111 ± 0.005 ***	0.021 ± 0.016 ***
10 μg/L TNF-α	0.519 ± 0.014	0.605 ± 0.020	0.314 ± 0.009
1 μM Mv-3-glc + TNF-α	0.341 ± 0.009 **	0.336 ± 0.021 **	0.183 ± 0.005 **
10 μM Mv-3-glc + TNF-α	0.175 ± 0.011 ***	0.261 ± 0.017 **	0.127 ± 0.001 ***
50 μM Mv-3-glc + TNF-α	0.039 ± 0.001 ***	0.098 ± 0.026 ***	0.049 ± 0.001 ***
100 μM Mv-3-glc + TNF-α	0.033 ± 0.002 ***	0.049 ± 0.001 ***	0.039 ± 0.001 ***
Control	0.023 ± 0.003 ***	0.102 ± 0.003 ***	0.013 ± 0.002 **
10 μg/L TNF-α	0.264 ± 0.009	0.337 ± 0.010	0.124 ± 0.006
1 μM Mv-3-gal + TNF-α	0.216 ± 0.010 *	0.208 ± 0.009 **	0.106 ± 0.001 *
10 μM Mv-3-gal + TNF-α	0.109 ± 0.001 **	0.185 ± 0.009 **	0.066 ± 0.001 **
50 μM Mv-3-gal + TNF-α	0.079 ± 0.001 **	0.133 ± 0.020 **	0.028 ± 0.003 **
100 μM Mv-3-gal + TNF-α	0.048 ± 0.001 ***	0.044 ± 0.022 ***	0.017 ± 0.001 **
Control	0.015 ± 0.003 ***	0.161 ± 0.005 ***	0.014 ± 0.001 ***
10 μg/L TNF-α	0.507 ± 0.011	0.625 ± 0.018	0.445 ± 0.005
1 μM (Mv-3-glc + Mv-3-gal) + TNF-α	0.421 ± 0.016 **	0.199 ± 0.008 ***	0.241 ± 0.004 ***
10 μM (Mv-3-glc + Mv-3-gal) + TNF-α	0.080 ± 0.002 ***	0.170 ± 0.001 ***	0.143 ± 0.004 ***
50 μM (Mv-3-glc + Mv-3-gal) + TNF-α	0.023 ± 0.001 ***	0.058 ± 0.005 ***	0.048 ± 0.003 ***
100 μM (Mv-3-glc + Mv-3-gal) + TNF-α	0.018 ± 0.001 ***	0.013 ± 0.002 ***	0.037 ± 0.002 ***

Note: *, **, and *** indicate *p* < 0.05, *p* < 0.01, and *p* < 0.001 respectively compared to TNF-α alone.

### 2.2. Effects of Mv-3-glc and Mv-3-gal on TNF-α-Induced ICAM-1 and VCAM-1 Proteins in Cell

**Figure 2:**
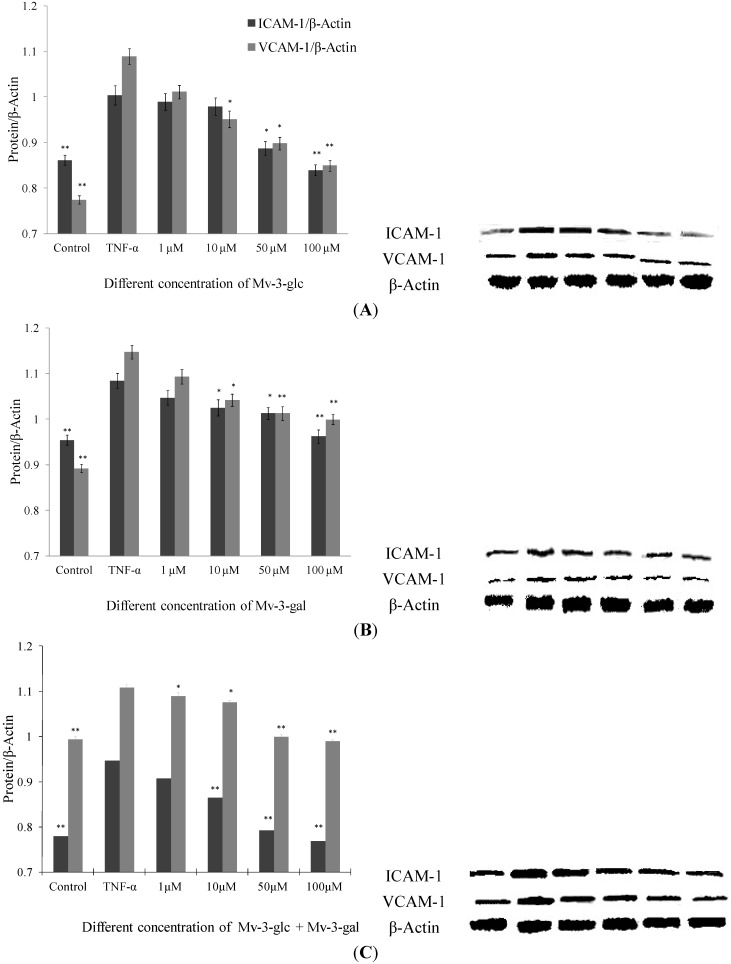
Effects of Mv-3-glc (**A**), Mv-3-gal (**B**), and their mixture (Mv-3-glc + Mv-3-ga) (**C**) on TNF-α-induced ICAM-1 and VCAM-1 protein expression. * and ** indicate *p* < 0.05 and *p* < 0.01 respectively compared to TNF-α alone.

Like supernatant, Mv-3-glc, Mv-3-gal and their mixture had different inhibitory effects on TNF-α-induced protein expression levels of endothelial ICAM-1 and VCAM-1 in a concentration-dependent manner (Figure 2). None of the samples had any significant inhibitory effects at a concentration of 1 μM, except for the mixture that inhibited increased VCAM-1 protein expression by 16.5% (*p* < 0.05). Since proteins in supernatant are secreted from cells, it indicated that malvidin glycosides affect more protein secretion inhibition than protein production. For most conditions, Mv-3-glc showed stronger effect than Mv-3-gal. Mv-3-glc at low concentration (1 μM and 10 μM) could not inhibit the increased ICAM-1 protein level, but at high concentration it showed significant inhibitory effects (for 50 μM, *p* < 0.05; and for 100 μM, *p* < 0.01, respectively). In addition, Mv-3-glc and Mv-3-gal at high concentration all inhibited increased ICAM-1 and VCAM-1 protein expression by more than 50%. Mv-3-glc at a concentration of 50 μM could inhibit 81.8% ICAM-1 and 54.6% VAM-1 (both *p* < 0.05), and Mv-3-glc at concentration of 100 μM could inhibited 115.4% ICAM-1 and 93.8% VCAM-1 (both *p* < 0.01); while 50 μM and 100 μM Mv-3-gal could inhibited 60.6% and 76.2% ICAM-1 (both *p* < 0.05), and 52.5% and 58.0% VCAM-1(both *p* < 0.01), respectively.

As to ICAM-1 level in cells treated with Mv-3-glc at concentration of 100 μM, and ICAM-1 and VCAM-1 protein expression levels in cells treated with 100 μM Mv-3-glc and Mv-3-gal mixture were less than the control (all *p* < 0.01), which also indicated their strong anti-inflammatory effects. Mv-3-glc had synergistic effect with Mv-3-gal, with their mixture’s inhibition rate was larger than that of each one, except for VCAM-1 expression in cells treated with the low concentrations of mixture (1 μM and 10 μM).

### 2.3. Effects of Mv-3-glc and Mv-3-gal on TNF-α-Induced MCP-1 and ICAM-1 mRNA in Cells

**Figure 3:**
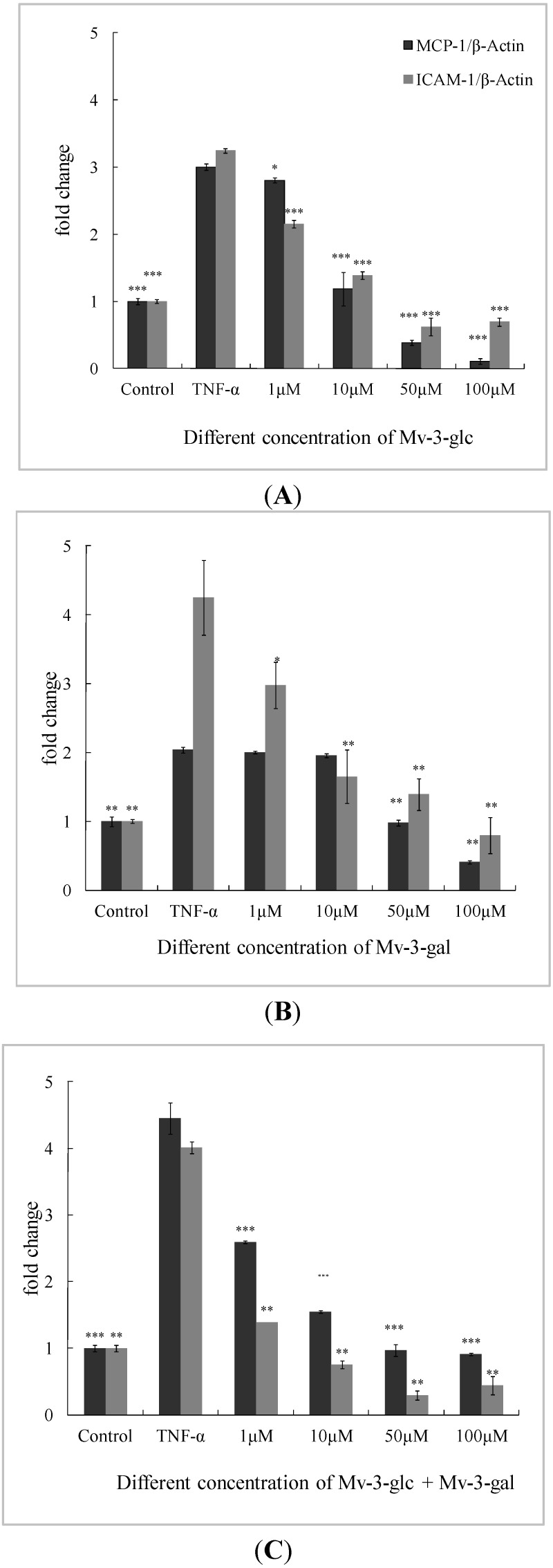
Effects of Mv-3-glc (**A**), Mv-3-gal (**B**), andtheir mixture (Mv-3-glc + Mv-3-ga) (**C**) on TNF-α-induced MCP-1 and ICAM-1 mRNA expression. *, **, and *** indicate *p* < 0.05, *p* < 0.01, and *p* < 0.001 respectively compared to TNF-α alone. β-Actin is used as internal standard, and mRNA levels are expressed as fold increase over the control.

Low concentration (1 μM) of Mv-3-gal had no significant inhibitory effect on MCP-1 mRNA expression. All the other treatments produced significant inhibition (*p* < 0.05) on MCP-1 and ICAM-1 mRNA expression in a concentration-dependent manner (Figure 3). 

Mv-3-glc exhibited better inhibitory effects than Mv-3-gal. Except for 1 μM (inhibition rate was 9.88%, *p* < 0.05), Mv-3-glc at all the other three concentrations had inhibition rates of more than 90% (*p* < 0.001) on MCP-1, and Mv-3-glc at all the four concentrations also had greatly significant inhibitory effects to ICAM-1 (*p* < 0.001). At the low concentration (1 μM), the inhibition rates of the mixture (53.8% for MCP-1 and 86.9% for ICAM-1) were much higher than Mv-3-glc (9.88% and 48.6%) and Mv-3-gal (3.04% and 39.2%), respectively. However, the mixture’s inhibition rates were not always more than each one for ICAM-1. High concentration of Mv-3-glc, Mv-3-gal, and their mixture expressed lower lever MCP-1 or ICAM-1 mRNA expressions than the control. These indicated that Mv-3-glc and Mv-3-gal could directly decrease MCP-1 and ICAM-1 mRNA expression without TNF-α-stimulation at the high concentration.

### 2.4. Effects of Mv-3-glc and Mv-3-gal on TNF-α-Induced IκB Degradation

**Figure 4:**
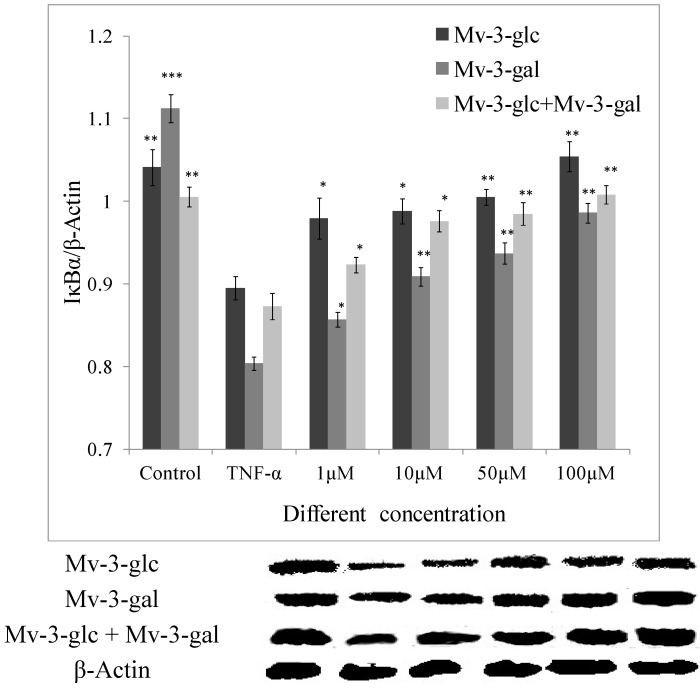
Effects of Mv-3-glc, Mv-3-gal, and their mixture (Mv-3-glc + Mv-3-ga) on IκBα protein levels in endothelial cells. *, **, and *** indicate *p* < 0.05, *p* < 0.01, and *p* < 0.001 respectively compared to TNF-α alone.

In this study, the amount of IκBα was greatly reduced after exposure to 10 μg/L TNF-α (*p* < 0.01). The inhibitory effect of Mv-3-glc, Mv-3-gal and their mixture at different concentrations on TNF-α-induced expression of IκBα degradation were all significant. They also were in the concentration-dependent manner. Mv-3-glc always exhibited better inhibitory capacity than Mv-3-gal in all the four concentrations. Mv-3-glc and Mv-3-gal mixture at the concentration of 10 μM and 50 μM had the inhibition rate of 78.0% and 84.8%, respectively, which were more than those of Mv-3-glc (63.7% and 75.3%) or Mv-3-gal (34.1% and 43.2%). Mv-3-glc and the mixture at the concentration of 100 μM could completely inhibit the IκBα degradation (*p* < 0.01), since the IκBα expression was even more than the control (Figure 4).

### 2.5. Effects of Mv-3-glc and Mv-3-gal on TNF-α-Induced NF-κB Translocation

**Figure 5:**
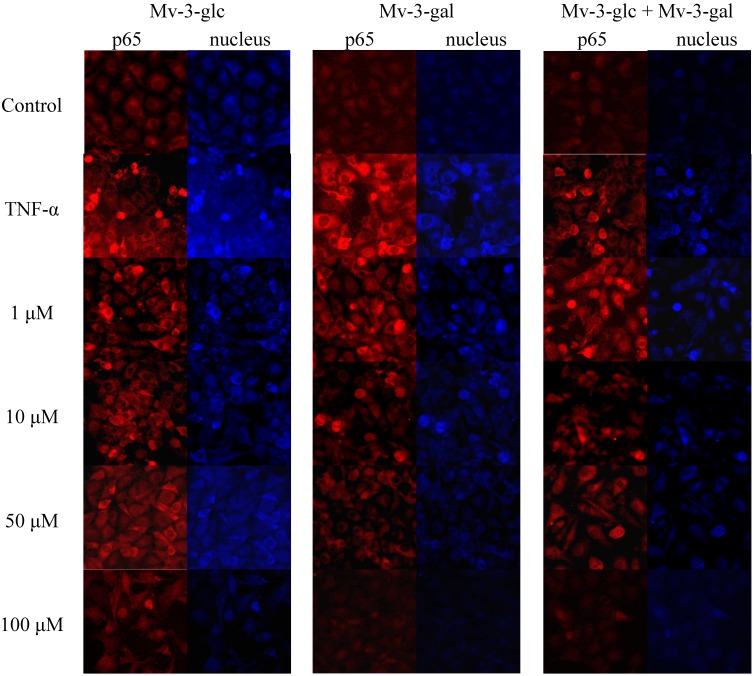
Effects of Mv-3-glc, Mv-3-gal, and their mixture (Mv-3-glc + Mv-3-ga) on NF-κB pathway (endothelial p65 translocation) in HUVECs.

On activation of the NF-κB pathway, the p65 protein is released from the cytosol and migrates into the cell nucleus where it interacts with the promoter regions of various proteins which up-regulated in inflammation [21]. Immunocytochemistry was performed by using NF-κB and fluorescein isothiocyanate (FITC)-conjugated antibody. In un-stimulated cells, the levels of p65 in the nucleus were very low. Upon simulation of TNF-α, the levels of p65 in the nucleus were increased. On the other hand, upon treatment of the cells with Mv-3-glc, Mv-3-gal, and their mixture, the fluorescence intensity levels of p65 were decreased in the nucleus (Figure 5). The high concentration of Mv-3-glc, Mv-3-gal, and their mixture inhibited the nuclear translocation of p65, with the p65 protein level in the cell nucleus similar to the control.

### 2.6. Discussion

Anthocyanins are one of the largest and most important groups of water-soluble pigments in most fruits. Berries, as colored fruits, are highly chemoprotective because of their bioactive anthocyanins [22,23]. Different anthocyanins offer different antioxidant capacity. A theoretical study evaluated the antioxidant character of three widespread anthocyanidins (cyanidin, delphinidin, and malvidin) [24] according to different parameters (bond dissociation enthalpy, ionization potential, proton affinity, and electron transfer enthalpy) and the atomic charges corresponding to the O atoms of the hydroxyl groups. It is found that antioxidant effect of anthocyanins is based on the free radical scavenging by means of the OH groups.

Wild lowbush blueberries (*Vaccinium angustifolium* Ait) are a rich source of anthocyanins and other flavonoids with anti-inflammatory activities, in which malvidin-3-glucoside was significantly more effective than epicatechin or chlorogenic acid in reducing the expression of pro-inflammatory genes *in vitro* [17]. In our studied Rabbiteye blueberry (*V. ashei*), Mv-3-glc and Mv-3-gal which are the two main glycoside forms of malvidin were the most abundant anthocyanins [15,25]. Malvidin possesses great antioxidant activity, and cytotoxicity against human monocytic leukemia cells and HT-29 colon cancer cells [26,27], and anti-hypertensive activity by inhibiting angiotensin I-converting enzyme (ACE) [28]. Our previous study reported the inhibitory effect of malvidin on TNF-α induced inflammatory response [16]. This study showed that Mv-3-glc and Mv-3-gal also possessed potential anti-inflammatory capacity. Mv-3-glc had better inhibitory effect than Mv-3-gal, indicating the differences in the two glycosides’ effects. In addition, Mv-3-glc and Mv-3-gal sometimes showed additive effects. An isobologram could be constructed in the future over a range of concentrations to verify whether they have synergy or not. Previous studies confirmed that Mv-3-glc could increase NO bioavailability, as well as inhibit peroxynitrite-induced NF-κB activation, which supported its benefits in cardiovascular health. A treatment of cyanidin 3-rutinoside and cyanidin 3-glucoside from the berry *Morus alba* L. also resulted in an inhibition on the activation of c-Jun and NF-κB, therefore it could decrease the *in vitro* invasiveness of cancer cells [29]. The anti-invasive activity on human colon cancer cells of the anthocyanins from fruits of *Vitis coignetiae Pulliat* was associated with modulation of constitutive NF-κB activation through suppression IκBα phosphorylation [30]. Lipopolysaccharide-induced NF-κB p65 translocation to the nucleus was markedly attenuated by anthocyanins of blueberry, blackberry, and blackcurrant, which mostly included malvidin-3-glucoside, cyanidin-3-glucoside and delphinidin-3-rutinoside. Their anti-inflammatory effects in macrophages were relative to their antioxidant capacity [31], so the antioxidant capacity made anthocyanins promising to develop nutraceuticals to improve endothelial function [32]. These indicate the anthocyanins could be the potential alternatives to prevent and treat the inflammation in many diseases [7]. Recent reports indicated action mechanisms of protecting vascular endothelium including modulation of crucial signaling pathways and gene regulation [33,34]. The present study showed that Mv-3-glc and Mv-3-gal also possessed anti-inflammatory properties by NF-κB pathway in endothelial cells.

Chronic inflammation is a common factor linking various pathologies in many diseases such as atherosclerosis and cancer. Vascular inflammation is a complex process, including the accumulation and activation of immune suppressor cells, pro-inflammatory cytokines, chemokines, growth and angiogenic factors and activation of several inflammatory signaling pathways mediated predominantly by NF-κB transcription factors [7]. The transcriptional activation of NF-κB plays a key role in the development of the inflammatory response [35]. It is well established that NF-κB is normally in an inactive form bound to inhibitory proteins, the IκBs. Exposed to external stimuli such as TNF-α, IKB kinase (IKK) phosphorylates IκBα, which lead to ubiquitination-dependent degradation of IκBα [36]. It is well accepted that the activation of NF-κB in endothelial cells is associated with mononuclear cell infiltration and an increased transcription of adhesion molecules, chemokines, and cytokines [21,37]. TNF-α can activate NF-κB, and then MCP-1, ICAM-1, and VCAM-1 were over expressed in vascular endothelial cells [38,39]. In this study, Mv-3-glc and Mv-3-gal inhibited TNF-α-induced MCP-1, ICAM-1 and VCAM-1 production as well as IκBα degradation. In addition, Mv-3-glc and Mv-3-gal were able to inhibit the nuclear translocation of p65, one subunit of NF-κB, suggesting the mechanism by which Mv-3-glc and Mv-3-gal could block pro-inflammatory signaling downstream of IκB.

## 3. Experimental Section

### 3.1. Chemicals and Reagents

Dulbecco’s PBS, M199 medium, TNF-α, trypsin, and Mv, Mv-3-glc, and Mv-3-gal were bought from Sigma Chemical Co., Ltd (Nanjing, China). Fetal bovine serum was purchased from Gibco/Invitrogen (Shanghai, China). Penicillin and streptomycin were obtained from Life Technologies (Shanghai, China). MCP-1, ICAM-1, and VCAM-1 ELISA Kit were purchased from Boster Biotechnology Inc. (Wuhan, China). Trizol reagent, PrimeScript RT master mix, and SYBR Green 2-step qRT-PCR kit were got from TaKaRa Bio Inc. (Dalian, China). NF-κB activation and nuclear translocation Assay Kit were bought from Beyotine Technology Inc. (Nanjing, China). All the chemicals and reagents were of the analytical grade.

### 3.2. Antibodies

Rabbit monoclonal primary antibody against ICAM-1, rabbit polyclonal primary antibodies against VCAM-1, mouse polyclonal primary antibody to the β-Actin antibody, and goat anti-rabbit/mouse HRP-conjugated secondary antibody, were purchased from Boster Biotechnology Inc. Rabbit monoclonal primary antibody to IκBα was bought from Beyotine Technology Inc. Primary antibodies were used at 1:200 dilutions, and secondary antibodies were used at 1:1000 dilutions.

### 3.3. Endothelial Cell Culture and Treatment

Human umbilical vein endothelial cells (HUVECs) are a representative model system for studying inflammation and oxidative stress in the vasculature [40]. HUVECs were saved in National Technical Research Centre of Veterinary Biological Products (Nanjing, China). The second to 6th passage cells were used for all experiments at 80%–90% confluence. HUVECs were quiesced in a reduced serum medium for 4 h prior to experiment. In a separate set of experiments, the cells were treated with 1, 10, 50, and 100 μM Mv-glc, Mv-gal, or their mixture for 18 h, followed by TNF-α (10 μg/L) stimulation for 6 h. DMSO was used as control. The supernatants were collected for ELISA analysis. The cells were prepared for western blotting.

### 3.4. ELISA Analysis and Western Blotting

The levels of MCP-1, ICAM-1, and VCAM-1 in the supernatants were quantified using ELISA kits. The assay procedure was employed according to the kit protocol booklet instructions. The absorbance of the resulting yellow color was measured at 450 nm on a StatFax-2100 Microplate Reader (Awareness Technology Inc., Palm City, FL, USA). The reader was controlled via Hyper Terminal Applet ELISA software. Western blotting was performed on the HUVEC lysates as described before [41]. Beside ICAM-1 and VCAM-1, IκBα was also analyzed by western blotting. Data were normalized by re-probing the membrane with an antibody against β-Actin which was used as a loading control.

### 3.5. Real-Time qRT-PCR

The total RNA was isolated from HUVECs using the Trizol reagent (TaKaRa Bio Inc.). It was reverse-transcribed into cDNA using PrimeScript RT master mix. For reverse-transcription, the SYBR Green 2-step qRT-PCR kit (TaKaRa Bio Inc.) was used. The real-time quantitative PCR analysis was carried out using the LightCycler 480 (Roche Diagnostics Inc., Rotkreuz, Switzerland). The primers for amplification were followed: forward primer 5-GTTGTCCCAAAGAAGCTGTGA-3 and reverse primer 5-AATCCGAACCCACTTCTGC-3 for MCP-1 (83 bp); forward primer 5-CCACAGTCACCTATGGCAAC-3 and reverse primer 5-AGTGTCTCCTGGCTCTGGTT-3 for ICAM-1 (124 bp); forward primer 5-TGGACTTCGAGCAAGAGATG-3 and reverse primer 5-GAAGGAAGGCTGGAAGAGTG-3 for β-actin (137 bp). The reaction was conducted with an initial denaturing at 94 °C for 30 s, then involved 40 cycles of 60 °C for 20 s, and at 65 °C for 15 s in the end. Relative gene expression data was analyzed using the 2^−^^ΔΔC^^t^ method.

### 3.6. Immunofluorescence

Nuclear translocation of p65 is widely used as a measure for NF-κB activation. HUVECs were fixed in 3.75% paraformaldehyde, and permeabilized with 0.1% Triton-X-100 incubated overnight with primary antibody against p65. On the following day, the cells were incubated with secondary antibody (goat anti-rabbit conjugated with FTTC) for 1 h. The cell nuclei were stained with DAPI (4',6-diamidino-2-phenylindole), and visualized under an Axiovision 4 Fluorescent Microscope (Zeiss, Oberkochen, Germany). All images presented are in (×100) magnification.

### 3.7. Statistical Analysis

All data presented are mean value ± standard deviation (SD). The data were analyzed by a one-way ANOVA using the SPSS 19.0 Statistical Software. Differences were considered significant with *p* value < 0.05.

## 4. Conclusions

In the present study, treatment with Mv-3-glc, Mv-3-gal, and their mixture significantly attenuated monocyte adhesion in TNF-α-stimulated HUVECs by inhibiting MCP-1, ICAM-1, and VCAM-1 protein and mRNA expressions both in endothelial cell and supernatants. In addition, they affected IκBα degradation and the nuclear translocation of p65, indicating that they possessed anti-inflammatory effects by blocking the NF-κB pathway mechanism. Mv-3-glc, Mv-3-gal, and their mixture all showed their inhibitory effects on TNF-α-induced inflammatory response in a concentration-dependent manner. Mv-3-glc had better potential anti-inflammatory effect than Mv-3-gal, and they showed synergistic effect sometimes. Blueberries are a good source of anthocyanins such as Mv-3-glc and Mv-3-gal, which can be a promising molecules for the development of nutraceuticals to improve endothelial function and thereby to prevent the progression of chronic inflammation.
